# Genomic supremacy: the harm of conflating genetic ancestry and race

**DOI:** 10.1186/s40246-022-00391-2

**Published:** 2022-05-19

**Authors:** Jessica P. Cerdeña, Vanessa Grubbs, Amy L. Non

**Affiliations:** 1 Yale School of Medicine, New Haven, CT 06510 USA; 2 Department of Ambulatory and Preventive Medicine, Alameda Health System, Oakland, CA 94602 USA; 3 Department of Anthropology, University of California San Diego, La Jolla, CA 92093 USA

**Keywords:** Genetic ancestry, Race, Disease-causing alleles, Structural racism

## Abstract

**Background:**

Recent studies have reignited the tinderbox of debate surrounding the use of race and ancestry in medicine. These controversial studies have argued for a strong correlation between genetic ancestry and race, justifying continued use of genetic ancestry measures in studies of disease. These studies contend that increased use of continental ancestry estimates can inform clinical risk assessments and management. Further, recent studies of racial corrections used in clinical algorithms, such as those used to estimate 'normal' lung function, also advocate for use of genetic ancestry in place of race for refining risk algorithms.

**Main body:**

These positions are misleading, harmful, and reflect superficial interpretations of population genetics. In this Perspective, we argue that continental genetic ancestry, often proxied by race, serves as a poor indicator of disease risk, and reinforces racialized inequities.

**Conclusion:**

Instead, we endorse that racial disparities in disease should be investigated by rigorous measures of structural racism alongside careful measures of genetic factors in relevant disease pathways, rather than relying on genetic ancestry or race as a crude proxy for disease-causing alleles.

## Background

In early 2021, the weekly medical journal, *The New England Journal of Medicine*, published two Medicine and Society articles that revived controversy over the use and value of race and genetic ancestry in medicine. The first, led by Borrell and colleagues, argues against what they term “a race/ethnicity-blind approach” and advocates for scholars and practitioners to consider “complementing the use of race/ethnicity with data on genetic ancestry, genotypes, or biomarkers” [1]. In a follow-up article, Oni-Orisan and colleagues affirm this stance, engaging their positionalities as Black geneticists. They argue for a “remarkably strong correlation between a person’s continent of ancestral origin and self-identified race” [2]. Both scholarly teams contend that increased use of continental ancestry estimates can inform clinical risk assessments and disease management. Additionally, these and other researchers have advocated for the use of genetic ancestry in place of race for refining disease risk algorithms, such as those used to estimate “normal” lung function [3].

In this Perspective, we argue that the positions advanced in these three recent papers reflect a troubling trend as they promote misleading, harmful, and superficial interpretations of population genetics. Continental genetic ancestry, sometimes proxied by race, serves as a poor indicator of disease risk, and reinforces ideas of genetically discrete racial groups, and leads, sometimes inadvertently, to racial bias in research and medicine [4]. Instead, we assert that racial disparities in disease should be investigated by rigorous and detailed measures of structural racism alongside appropriate measures of genetic factors in relevant disease pathways—which may both independently or through interactions—explain portions of disease risk, rather than relying on genetic ancestry or race as a crude proxy for disease-causing alleles.

## Main text

### Race and continental ancestry

Invented biological racial types emerged during the convergence of the eras of Enlightenment and Imperialism, engaging pseudoscientific language and methods to rationalize the global subjugation of Black and Brown populations [5]. Early iterations of race taxonomized humans based on continental origins and ascribed phenotypic, behavioral, and biological traits to each group based on flawed evidence [6, 7]. This approach vastly underestimates genetic diversity within continental boundaries and the extent of gene flow across continental groups. For instance, a study that Oni-Orisan and colleagues cite as evidence of correlation between self-described race and genomic ancestry defined a set of predefined single nucleotide polymorphisms (SNPs) of interest based on participants’ self-assigned race or ethnicity, which the authors collapsed from 23 classifications into seven: “East Asian,” “Pacific Islander,” “Latino,” “African descent,” “White European,” “South Asian,” and “Native American” [8]. This over-simplification of racial diversity led to artificially inflated associations between race and genetic ancestry. In addition, the authors found considerable overlap between Asian and European populations, extensive “admixture” of European and African ancestry with African ancestry ranging from 10.6 to 100 percent among the “African descent” population, nearly indistinguishable overlap of “Latino” participants with other groups, as well as broad variation of continental ancestry proportions [8]. The authors summarize their findings by noting the high correlations of participants who self-defined their race or ethnicity and demonstrated at least 5 percent of ancestry from a specific continent of origin. In brief, these data do not meaningfully corroborate assertions of high correspondence between continental ancestry and self-described race.

The confusion over the meaning of race and genetic ancestry is further exacerbated by direct to consumer (DTC) genomic testing companies, which sell genetic ancestry tests that typically provide oversimplified and misleading results that reify biological concepts of race. Several companies promise to deliver information on a consumer’s heritage, race, or ethnicity, while instead identifying relatively broad continental ancestry groups. Further, results reports often fail to explain the limitations of these estimates, which are based on self-reported geographic location of currently living (i.e., not ancestral) individuals, and rely on the limited diversity of the reference database and the varied algorithms used to estimate the results [9, 10]. For instance, 23andMe clusters its “ancestry composition” according to conventional racial categories, including “European” and “Sub-Saharan African”—which commonly describes dark-skinned Africans—but does not distinguish between race and genetic ancestry. Other companies (e.g., Genelex, Niagen, Genetree) have advertised genetic markers unique to Native Americans; however, these purported markers are also found in smaller percentages in other populations [11].

### Genetic ancestry and disease risk

Continental ancestry cannot appropriately contribute to assessments of disease risk. First, most human genetic diversity results from random mutation and serial founder events due to geographic and reproductive isolation that are not bound by continental borders [12, 13]. Second, most complex diseases exhibit gene-by-environmental interactions and the confluent effect of susceptibility alleles across multiple loci and thus ancestral patterns of genetic drift or local regional selection should be less relevant in these diseases [14]. Third, the greatest degree of human genetic diversity occurs within the continent of Africa [15], and so estimates of “African ancestry” overlook variation throughout the continent. Finally, global patterns of migration and admixture erode the utility of continental ancestry as a predictor of genotype and attendant disease risk [8].

Consider the popular example of sickle cell disease (SCD). Although clinicians frequently conflate Black race—or “African ancestry”—with risk of SCD, the distribution of the variant that gives rise to the condition occurs inequitably across the African continent as well as in areas of the Middle East, South Asia, and Latin America. Moroccans and Algerians enjoy little to no risk of carrying the variant, whereas the frequency is much higher in west-central African countries like Nigeria, Cameroon, and Angola [9]. In fact, SCD haplotypes also lack homogeneity even within Africa, with at least four distinct haplotypes of potentially varying severity differentially distributed throughout the continent [16]. Furthermore, SCD is an exception, a Mendelian disease driven by a single point mutation that emerged under selective pressure, a less common source of genetic variation in humans. Diseases more commonly identified in assessments of racialized health disparities, such as diabetes and cancer, demonstrate poly- and omnigenic influences as well as plastic responses to environmental conditions patterned by structural racism, including nutrition and toxic exposures [10]. Thus, continental ancestry has limited utility for prediction of risk for these common and complex diseases.

Clinicians and researchers often debate over the utility and precision of terms such as “race,” “ethnic group,” “genetic ancestry,” and “population.” Often, scholars use these terms interchangeably without defining them; as such, “race” and “ethnic group” carry inferred biological significance, despite consensus that race represents a hierarchical ordering of humans according to shifting sociopolitical conditions and “ethnic group” refers to communities that share cultural or linguistic characteristics distinct from dominant social groups [17]. Although many presume “genetic ancestry” can more accurately substitute for “race” or “ethnic group” as it refers to the geographic residence of a person’s ancestors, the term is laden with the ambiguities we described above. Like ancestry, “population” may appear to be a more neutral and value-free descriptor; however, “population” is vague and subject to imbuement with typological and essentialist assumptions that surround terms like “race” [18]. We recommend researchers use specific terms about the geographic origin of the individual, beyond continent or sub-continent—such as Igbo instead of sub-Saharan African—and emphasize the need to test for genetic risk alleles rather than presuming patients from certain “races,” “ethnic groups,” or “populations” carry them by default [19].

### Genetic ancestry and disease risk algorithms

Some researchers have argued that genetic ancestry is more precise than self-identified racial categories and should be used in place of self-identified race in algorithms that estimate disease risk [2, 11]. For example, a recent study of reference equations used in estimating lung function in “admixed” African American and Puerto Rican children argues for incorporation of genetic ancestry estimates into spirometry reference equations to improve precision of equations that currently use a racial correction for non-White groups [3]. However, beyond the infeasibility for clinicians to gather genetic ancestry data in a routine clinical setting, we argue this is a dangerous recommendation. First, because it implies genetically meaningful differences between racial groups that are relevant to lung function, which have not been demonstrated, and thus incorrectly reifies race as a biological concept. Second, these ancestry estimates are treated as more objective than self-identified race but are limited in accuracy by reliance on contemporary reference populations, and don’t necessarily capture any markers of interest relevant to disease risk [6]. Third, and most importantly, this recommendation ignores the possibility that ancestry differences may reflect exposures to different environments across racial groups that cause real damage to lungs that is otherwise ignored in a race-adjusted equation. Consider that a person who identifies as Black may have 75% “African” genetic ancestry and deeply pigmented skin, the latter of which is linked with a legacy of structural discrimination in the United States, making them more likely to be exposed to toxic air pollutants in their neighborhoods or occupations. By including an African ancestry component in the reference equation for “normal” lung function, clinicians assume they are adjusting for genetically meaningful differences in lung function by ancestry, but may actually normalize true lung impairment resulting from environmental racism. In the absence of specific knowledge of contributory genetic loci that vary by race or ancestry, including either category in clinical algorithms risks exacerbating rather than reducing racial health disparities [20].

### The harm of geneticizing race

Races are not biological categories that can be discerned by genetic frequencies. Instead, race is a product of racist ideology that situates White people above Black and Brown people. Deploying genetic underpinnings for race serves to legitimize practices of White supremacy and distracts from structural sources of racial health inequities [21]. As epidemiologists Cooper and David argued nearly four decades ago, presuming racialized health disparities arise from genetic causes “accepts as given precisely the thing to be explained” [22]. In other words, we should not merely accept racial or ethnic health disparities as the consequence of genomic ancestry without specific genetic evidence. Nor should we vaguely attribute inequality to social environment differences. Instead, we should rigorously investigate these contributions through measurement of the health consequences of structural racism—including assessment of educational and employment inequities, interactions with police and the judicial system, and residential segregation—or, when appropriate, targeted genetic testing unbiased by racial classification.

If we recognize that racial disparities result, at least in part, from experiences of racism, we should expand our efforts to investigate and intervene upon these harmful social structures. The use of racial self-identification as a proxy for “racism” is too simple to provide useful information as it assumes a false narrative that all people of a given race have a single exposure and perception of racism. Rather, several research groups have launched approaches to operationalizing structural racism that can inform future research [23]. In addition, evaluation of the impacts of individual dimensions of structural racism—such as inequities in wealth accumulation and work conditions—can yield more specific insight regarding policy intervention. Attributing these differences to genetics misguides researchers and clinicians to seek pharmacologic interventions—a largely unfruitful tactic [24, 25]—or, worse, excuses a “do-nothing” approach based on the belief that genetic disease risk cannot be modified. Racialized health inequities result from multiple modifiable conditions that can be ameliorated through policy action including desegregation, housing support, anti-discrimination policies, and reparations.

## Conclusions

The inappropriate conflation of race with genomic ancestry buttresses architectures of White supremacy, exacerbates racism in medicine, and exculpates policymakers. Genomics research should rely on whole-genome sequencing to identify genetic associations with outcomes of interest, rather than imposing selective SNP testing based on social categories, whether self- or investigator-assigned. Further, we argue genetic ancestry should not be used in place of race in clinical risk algorithms, as it is conflated with social and environmental conditions and may lead to inaccurate risk assessment. At the same time, the biomedical community should expand its efforts to identify intervenable instances of structural racism on racialized health inequities and advocate for policy reform to improve population health.

## Data Availability

Not applicable.
